# Soft tissue calcifications: a pictorial essay

**DOI:** 10.1590/0100-3984.2019.0100

**Published:** 2020

**Authors:** Luisa Leitão de Faria, Fernanda Babler, Lorena Carneiro Ferreira, Ozeas Alves de Noronha Junior, Felipe Lorenzo Marsolla, Dalton Libânio Ferreira

**Affiliations:** 1 Hospital das Clínicas da Faculdade de Medicina da Universidade de São Paulo (HC-FMUSP), São Paulo, SP, Brazil.

**Keywords:** Calcinosis/diagnostic imaging, Radiography, Diagnosis, differential, Calcinose/diagnóstico por imagem, Radiografia, Diagnóstico diferencial

## Abstract

Soft-tissue calcifications are extremely common. Because the imaging findings are nonspecific, soft-tissue calcifications are often problematic for radiologists, sometimes prompting unnecessary interventions. In addition, the nomenclature is quite confusing. Classically, soft-tissue calcifications are divided into four categories, by mechanism of formation-dystrophic, iatrogenic, metastatic, and idiopathic-depending on the clinical and biochemical correlation. However, it is also possible to classify such calcifications by compartment, and that classification can be quite useful in the radiological diagnostic assessment. In this article, we illustrate the main causes of soft-tissue calcifications, organizing them according to their anatomical and pathophysiological aspects, thus narrowing the differential diagnosis.

## Abstract (pt)

Calcificações de partes moles são achados extremamente comuns e inespecíficos nos exames de imagem e, por isso, frequentemente são fonte de confusão por parte dos radiologistas, desencadeando, por vezes, intervenções desnecessárias. Além disso, a nomenclatura atribuída é muito confusa. Classicamente, dividem-se as calcificações de partes moles, conforme seu mecanismo de formação, em calcificações distróficas, iatrogênicas, metastáticas e idiopáticas, dependendo de correlação clinicolaboratorial, porém, também é possível uma classificação compartimental das calcificações, que pode ser muito útil na propedêutica radiológica. Neste trabalho, ilustramos didaticamente as principais causas de calcificações de partes moles organizando-as de acordo com aspectos anatômicos e fisiopatológicos, estreitando os diagnósticos diferenciais.

## INTRODUCTION

On imaging examinations, soft-tissue calcifications are findings that are as common as they are nonspecific, ranging from a nonspecific local reaction (in response to a traumatic insult) to the manifestation of a systemic condition^(1,2)^. In addition to this lack of specificity and the long list of differential diagnoses, the nomenclature is confusing and not very intuitive^(3,4)^.

Classically, these calcifications are divided into four categories, depending on the mechanism of formation as well as the clinical and biochemical correlation^(5)^: dystrophic, metastatic, idiopathic, and iatrogenic. However, it is also possible to classify such calcifications by compartment (subcutaneous, neurovascular, fascial, muscle, and periarticular), and that classification can be quite useful in the radiological diagnostic assessment^(1,2)^.

In this article, we illustrate the main causes of soft-tissue calcifications in a practical and didactic manner. We believe that this knowledge will help narrow the differential diagnosis of such calcifications.

## DYSTROPHIC AND IATROGENIC CALCIFICATIONS

Dystrophic and iatrogenic calcifications occur in damaged or degenerated tissue and account for 95-98% of all soft-tissue calcifications^(1,2)^. Dystrophic iatrogenic calcifications are correlated with surgical manipulation or medication infusion^(6)^. They can vary in size and shape and are not usually related to any systemic metabolic abnormality^(2,6)^. In some cases, these damaged tissues go through a process of ossification, characterized by the formation of cortical and medullary tissue^(4)^. The first step in assessing dystrophic calcifications is to determine their location^(2)^.

### Vascular compartment

**Figure 1:**
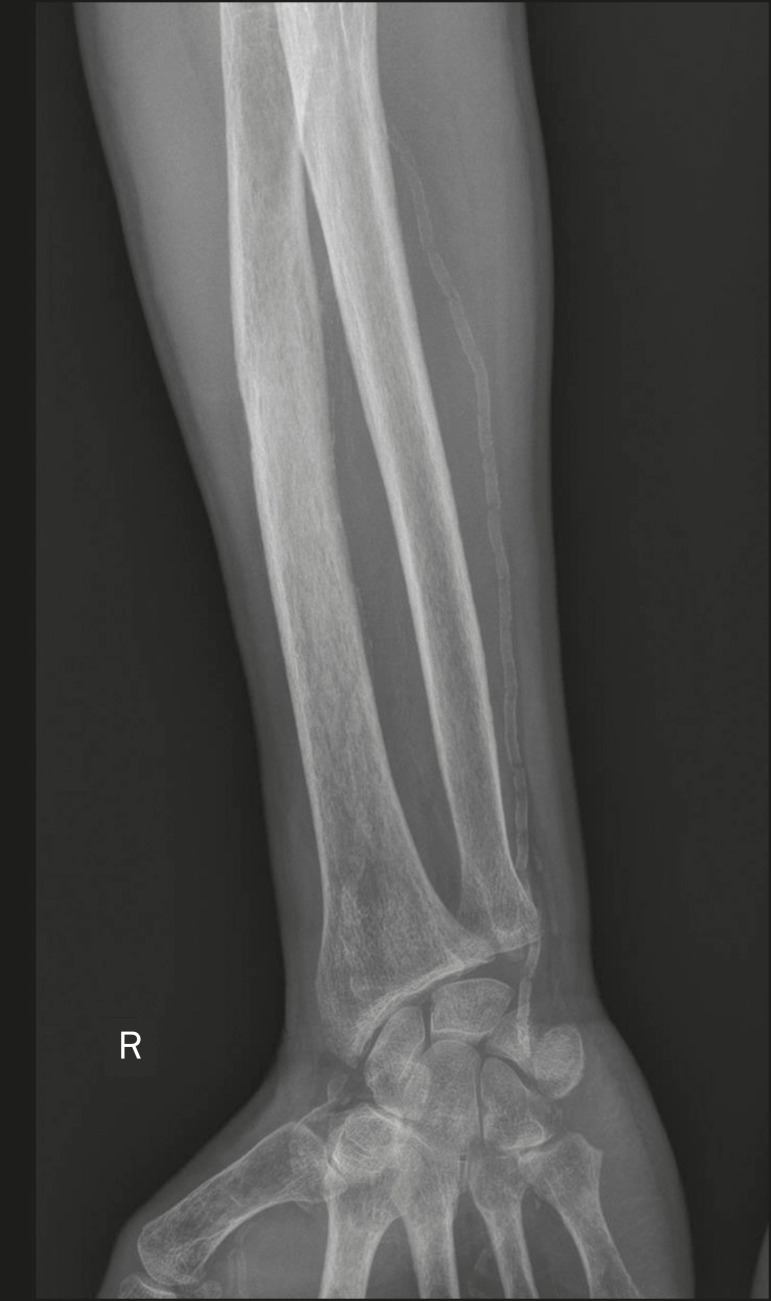
Vascular calcifications in medial calcific sclerosis (Mönckeberg’s arteriosclerosis). Frontal radiograph of the forearm showing small, diffuse, circumferential calcifications in the ulnar artery wall and no luminal narrowing, which is characteristic of Mönckeberg’s arteriosclerosis, caused by calcium deposition in the middle layers of small- and medium-caliber vessels.

**Figure 2:**
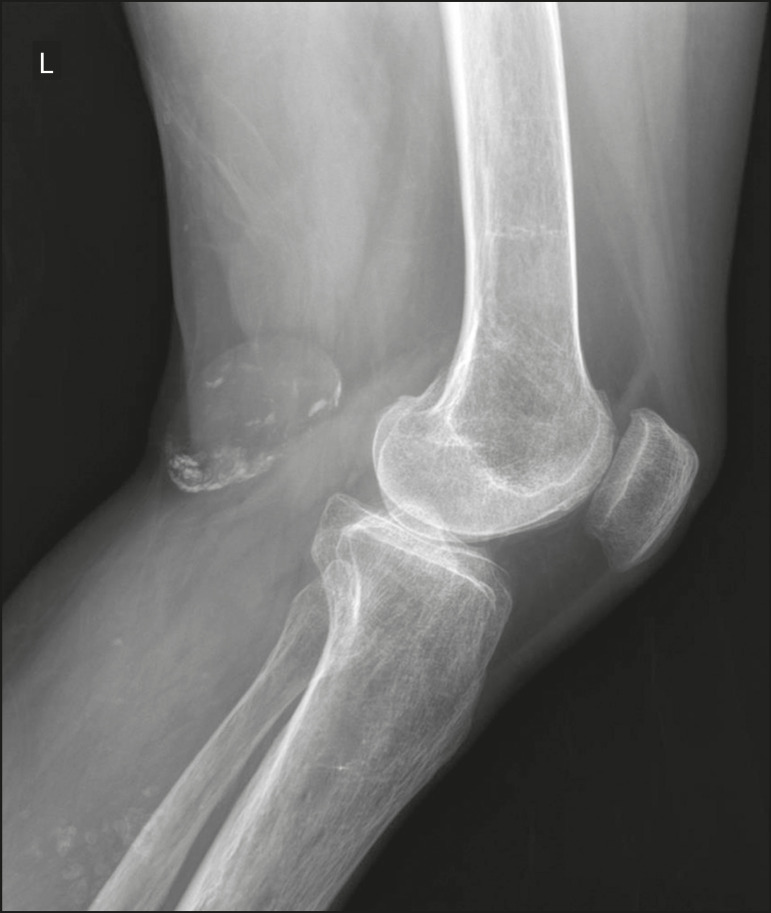
Popliteal artery aneurysm. Lateral X-ray of the knee showing peripheral oval-shaped calcifications along the course of the popliteal artery, which presents dilation consistent with an aneurysm.

Vascular calcifications are calcifications of the vessel walls, which are, therefore, distributed in neurovascular compartments. They can occur in various conditions^(1,6)^: atherosclerotic disease; diabetes mellitus, which can lead to Mönckeberg’s arteriosclerosis (Figure 1); aneurysm (Figure 2); chronic venous insufficiency; and venous malformations, from which phleboliths can arise. Vascular calcifications have a characteristic shape, being either tubular or arranged in parallel lines^(1,2,6)^.

### Subcutaneous compartment

Subcutaneous calcifications are associated with diseases of various origins. Preeminent among those diseases are conditions with inflammatory and traumatic causes, such as connective tissue diseases, injection-site granulomas, and panniculitis ossificans, which is a form of heterotopic ossification involving the subcutaneous tissue, usually resulting from local trauma^(1)^.

### Fascial compartment

**Figure 3:**
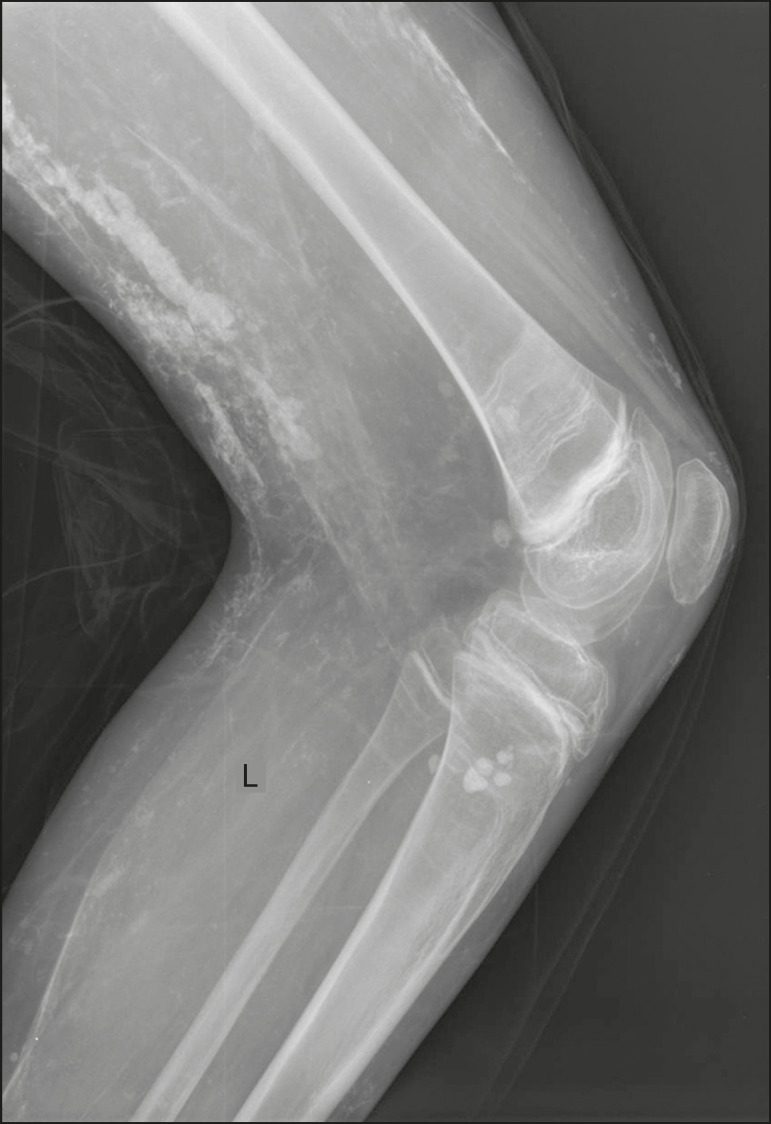
Fascial calcifications in dermatomyositis. Lateral X-ray of the knee of a patient with dermatomyositis, showing leaf-like calcifications along the fascial planes and subcutaneous tissue, outlining the muscle groups

Calcifications of the fascial compartment are associated with dermatomyositis and polymyositis, diseases that are characterized by muscle inflammation, with or without cutaneous/subcutaneous involvement, a clinical feature that differentiates them from other conditions. During the chronic phase of these diseases, after episodes of myositis, calcifications develop in necrotic areas of the fascial planes. The calcifications are typically long and linear (leaf-like), following the outline of the fascial compartment^(1)^, as depicted in Figure 3.

### Muscle compartment

**Figure 4:**
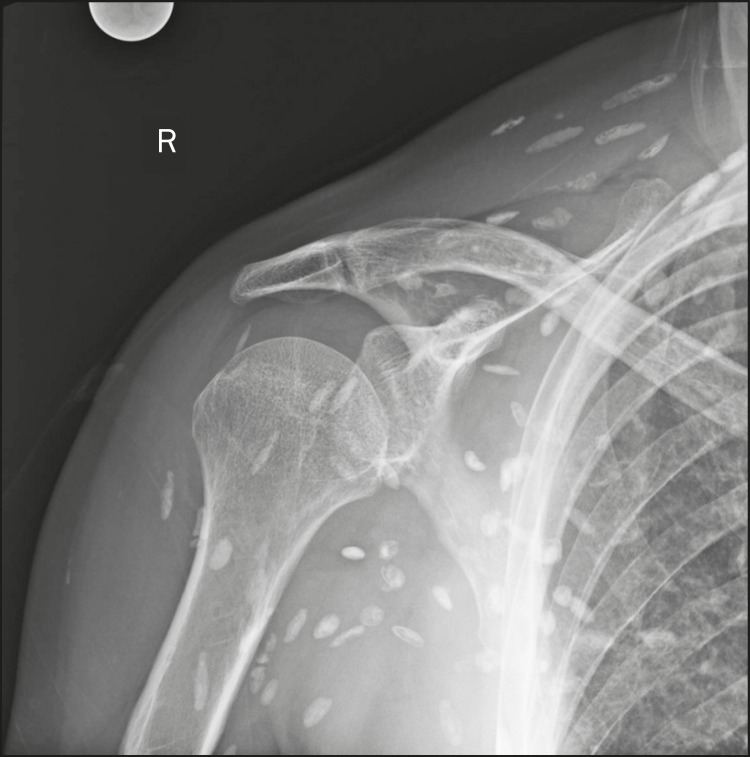
Muscle calcifications in cysticercosis. Frontal X-ray of the shoulder showing multiple dystrophic calcifications diffusely distributed in the muscle bellies, with a typical rice-grain-like morphology.

**Figure 5:**
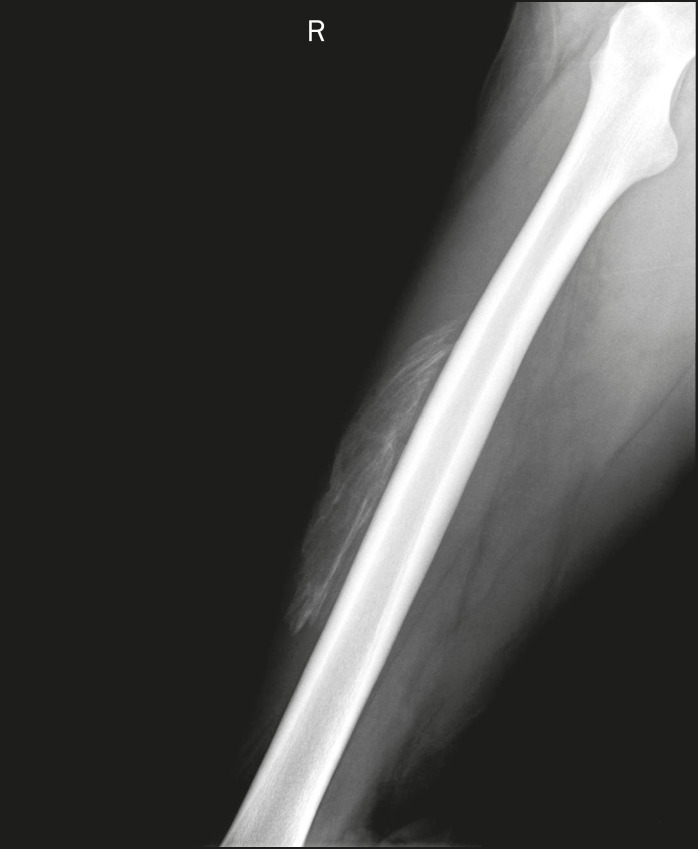
Muscle calcifications in myositis ossificans. Lateral X-ray of the thigh showing calcifications with cortical and medullary spaces outlining the fibers of the muscle belly.

**Figure 6:**
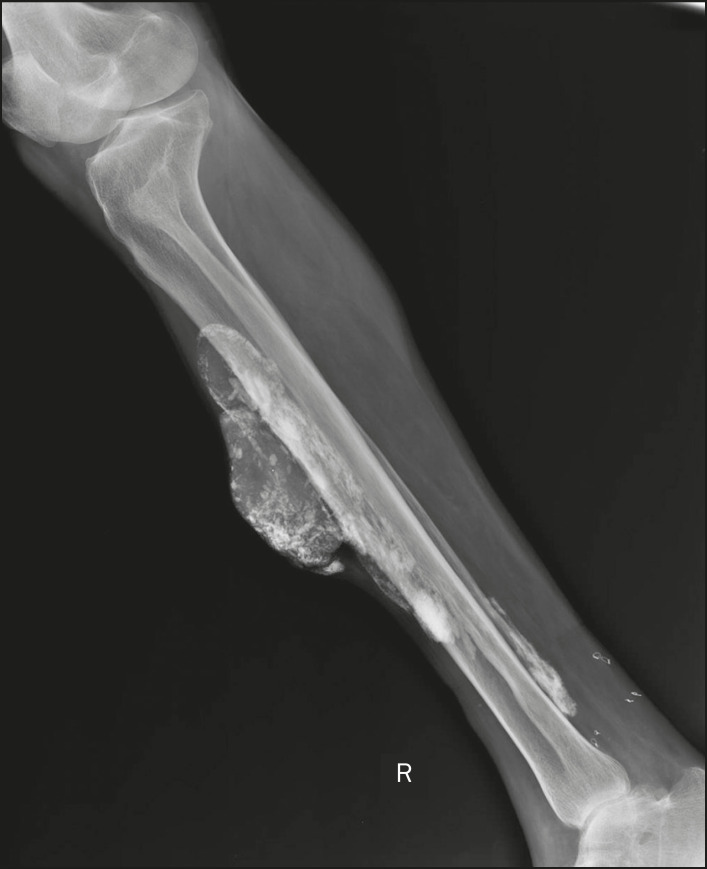
Muscle calcifications in calcified muscle hematoma. Lateral X-ray of the leg showing a mass with predominantly peripheral soft-tissue calcifications, corresponding to a muscle hematoma with dystrophic calcifications.

**Figure 7:**
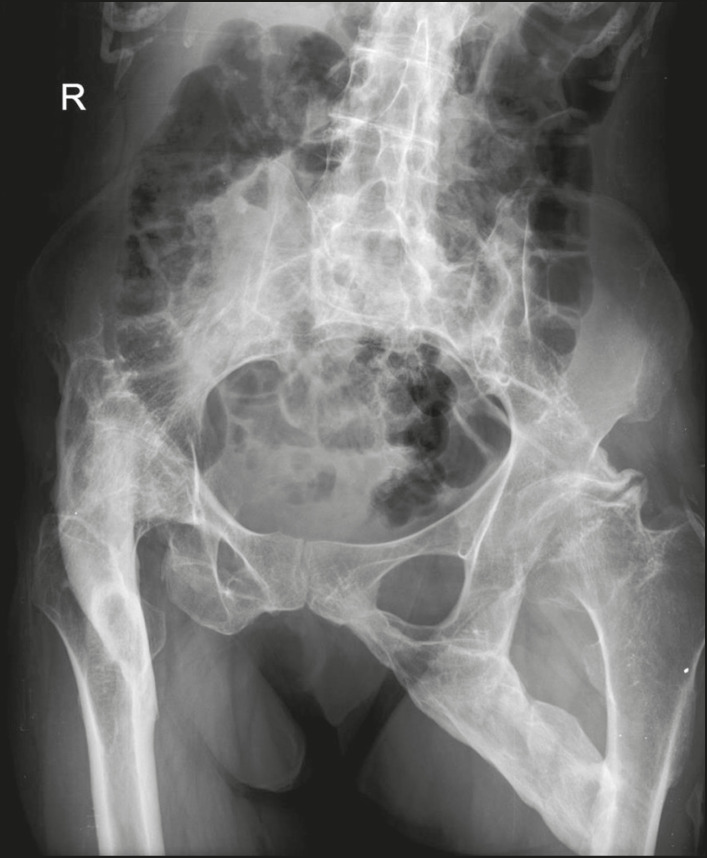
Muscle calcifications in fibrodysplasia ossificans progressiva. Frontal X-ray of the pelvis showing heterotopic ossification between the pelvis and the femur in a patient with fibrodysplasia ossificans progressiva evolving to restriction of lower limb mobility.

Calcifications in the muscle compartment result from infectious, traumatic, or congenital insults^(1)^. Preeminent among the infectious causes are granulomatous and parasitic infections, particularly cysticercosis (Figure 4). Chief among the traumatic causes is myositis ossificans (Figure 5), a type of heterotopic ossification that develops after a trauma (burns are a classic cause). In individuals with myositis ossificans, the initial X-ray findings (small calcifications) appear two to six weeks after the trauma^(6)^. Although it is called myositis, there is no inflammatory process^(1,6)^. Other traumatic causes of muscle calcifications include calcified muscle hematomas (Figure 6) and calcific myonecrosis^(4)^. The congenital causes include fibrodysplasia ossificans progressiva (Figure 7), a rare autosomal dominant disorder that is extremely debilitating, characterized by diffuse, progressive heterotopic ossification and equally progressive impairment of mobility^(7)^.

### Periarticular compartment

**Figure 8:**
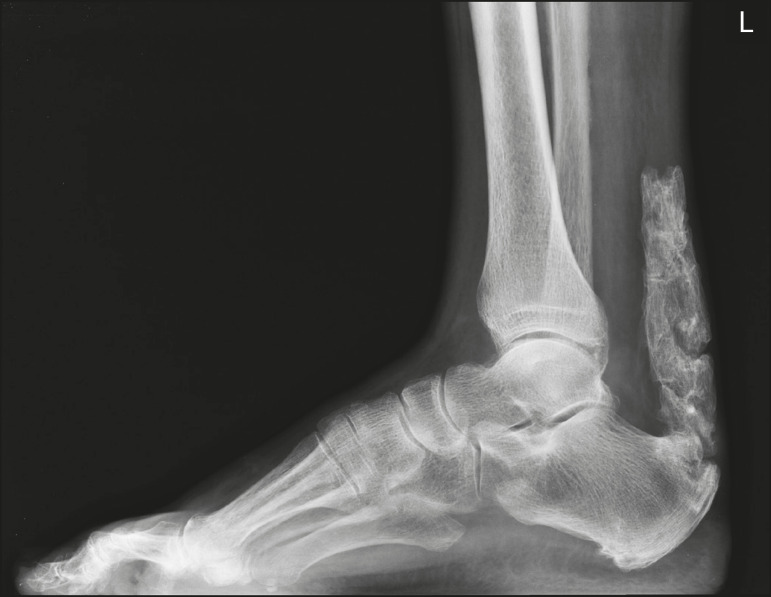
Ossification of the calcaneal tendon. Lateral X-ray of the ankle showing ossification of the calcaneal tendon after surgery to repair a tear, with an organization typical of mature bone (cortical and medullary spaces).

Periarticular calcifications occur in intra- or extra-articular components that have previously been involved in inflammatory or degenerative processes. Extra-articular calcifications occur when in tendons, bursae, and ligaments (Figure 8), typically after surgery/trauma or as a result of hydroxyapatite crystal deposition.

**Figure 9:**
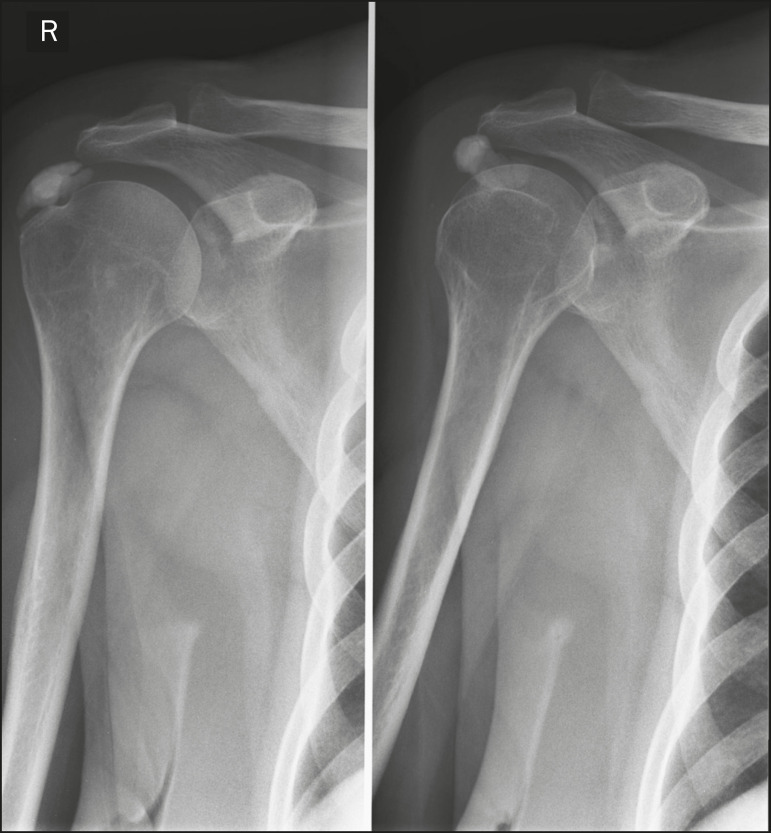
Calcific tendinopathy (hydroxyapatite deposition). Frontal X-ray of the shoulder showing nodular calcifications accumulating in the supraspinatus tendon.

**Figure 10:**
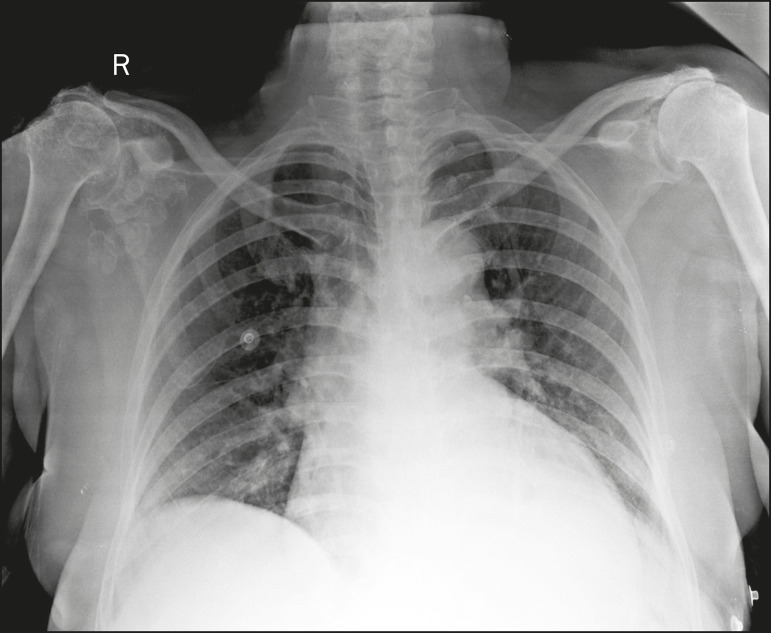
Hydroxyapatite-induced arthropathy in Milwaukee shoulder syndrome. Frontal X-ray of the chest showing the right shoulder with joint surface deformity, joint space reduction, and superior displacement of the humeral head with loss of the subacromial space (probably associated with a rotator cuff tear). Periarticular calcifications and intra-articular loose bodies can also be seen.

As shown in Figure 9, calcific tendinopathy and calcific bursitis (hydroxyapatite deposition in degenerated tendons or bursae) are quite common in the general population, usually affecting the shoulder (the tendon of the supraspinatus muscle) in the fifth decade of life^(2,8)^. In addition to their deposition in the tendons, the hydroxyapatite crystals can accumulate in the joints (crystal arthropathy), causing synovitis and articular damage. The most commonly affected site is the shoulder, causing what is known as Milwaukee shoulder syndrome^(1)^, as depicted in Figure 10.

**Figure 11:**
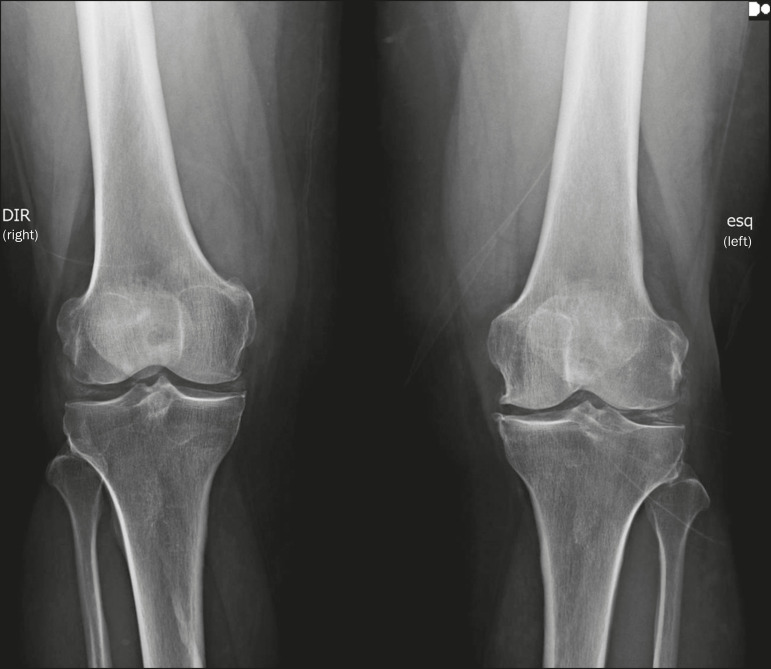
Articular cartilage calcifications in CPPD deposition disease. Frontal X-ray of the knee showing small calcifications in the articular hyaline cartilage and in the meniscus. Note the linear, stratified aspect of the calcifications.

Intra-articular calcifications are calcifications of articular cartilage (chondrocalcinosis). The most common cause of such calcifications is arthropathy resulting from the deposition of calcium pyrophosphate dihydrate (CPPD) in hyaline cartilage and fibrocartilage, including the meniscus, acetabular labrum, and intervertebral discs^(3,6)^, as illustrated in Figure 11. The term chondrocalcinosis denotes the radiological or histological identification of calcifications in cartilage, which can occur in metastatic calcifications; therefore, it should not be used as a synonym for CPPD deposition disease. Another term that is often used incorrectly to refer to CPPD deposition disease is pseudogout, which actually refers to a gout-like clinical syndrome and not to the radiological finding^(3)^. The origin of CPPD arthropathy is most probably degenerative, resulting from an abnormality in the local metabolism of the synovial fluid and articular cartilage^(2)^. It is quite common in the elderly and is usually asymptomatic^(3)^.

The shape and distribution of intra-articular calcifications facilitate their differential diagnosis. Calcifications resulting from hydroxyapatite deposition (calcific tendinopathy and calcific bursitis) are nodular or cotton ball-like, mainly affecting extra-articular tissue^(8)^, whereas those resulting from CPPD deposition tend to be smaller and more linear, with a stratified appearance^(2)^.

**Figure 12:**
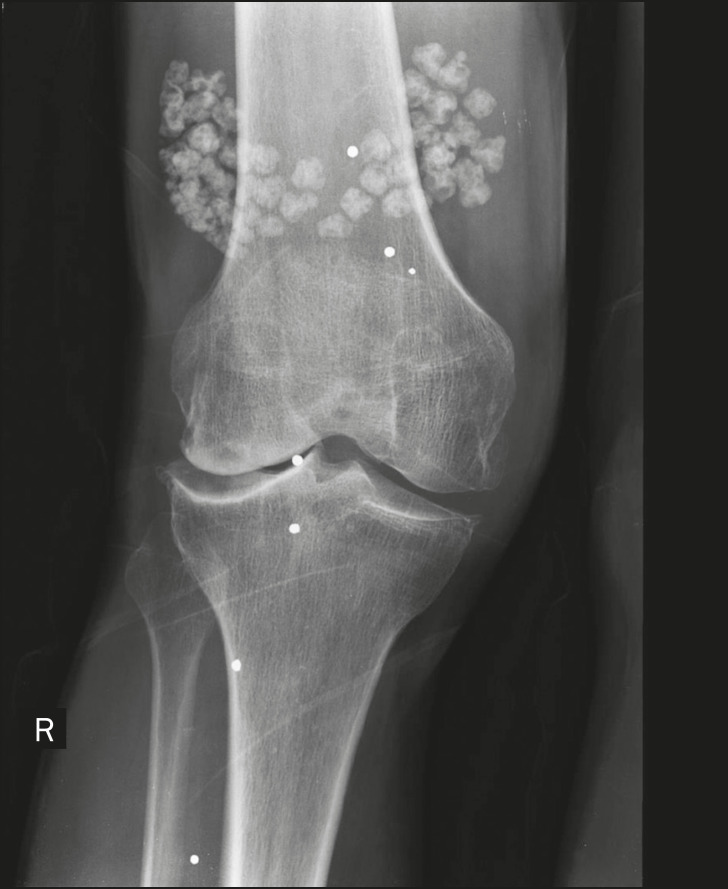
Synovial osteochondromatosis. Frontal X-ray of the knee. The radiographic appearance is often diagnostic, showing numerous rounded calcified bodies within the joint space, usually measuring 1–20 mm in diameter, accompanied by joint effusion.

Another cause of intra-articular calcification is synovial osteochondromatosis, a rare entity caused by chondroid metaplasia of the synovial tissue with proliferation of osteocartilaginous bodies within the synovium. These bodies typically have a chondroid mineralization pattern and can be completely calcified or have a typical calcified halo^(1,4,9)^, as depicted in Figure 12.

Various other benign and malignant tumors of bone or soft tissue produce periarticular (intra- and extra-articular) calcifications, including a wide range of differential diagnoses^(1,6)^, the review of which is beyond the scope of this article.

## METASTATIC (METABOLIC) CALCIFICATIONS

Metastatic (metabolic) calcifications are generalized calcifications that occur in normal tissue and are typically caused by the deposition of calcium salts resulting from a systemic metabolic disorder that leads to an elevation of the calcium-phosphate product to above the 60-70 range^(2,4)^.

**Figure 13:**
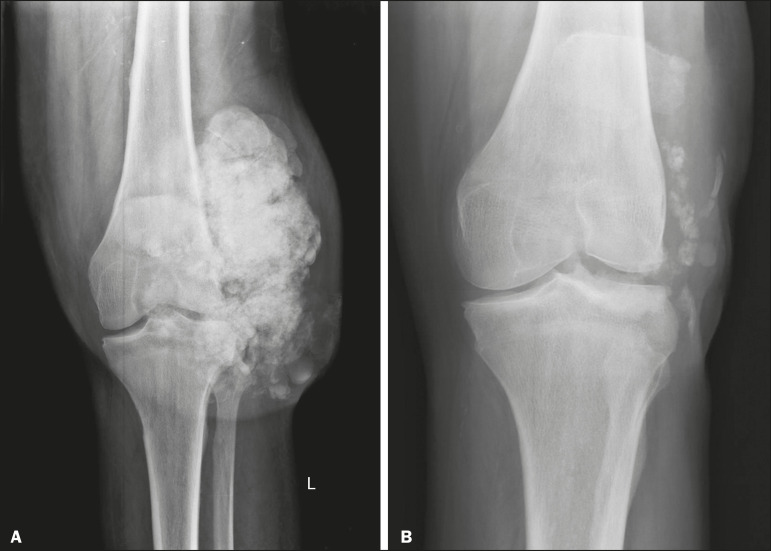
Metastatic calcifications in end-stage renal disease. **A:** Frontal X-ray of the knee showing a periarticular calcified mass with lobulated contours on the lateral face of the knee, with a multicystic appearance and a fluid-calcium level (the sedimentation sign). Note that the radiological appearance is identical to that of tumoral calcinosis. **B:** Frontal X-ray of the knee two years after kidney transplantation showing significant shrinkage of the lesions.

The most common cause of metastatic calcifications is end-stage renal disease, which is also the main cause of massive periarticular calcifications. When that is the case, the metabolic disorder results from renal dysfunction, showing a correlation with the duration of the disease and possibly occurring even in the absence of hyperparathyroidism^(4,9)^. Metastatic calcifications consist of periarticular lobulated calcified masses that are usually multicystic and contain fluid-calcium levels (Figure 13), which is known as the sedimentation sign^(1,4,10)^ . Other causes of metastatic calcifications with disorders of calcium and phosphate metabolism include primary hyperparathyroidism, milk-alkali syndrome, and hypervitaminosis D.

**Figure 14:**
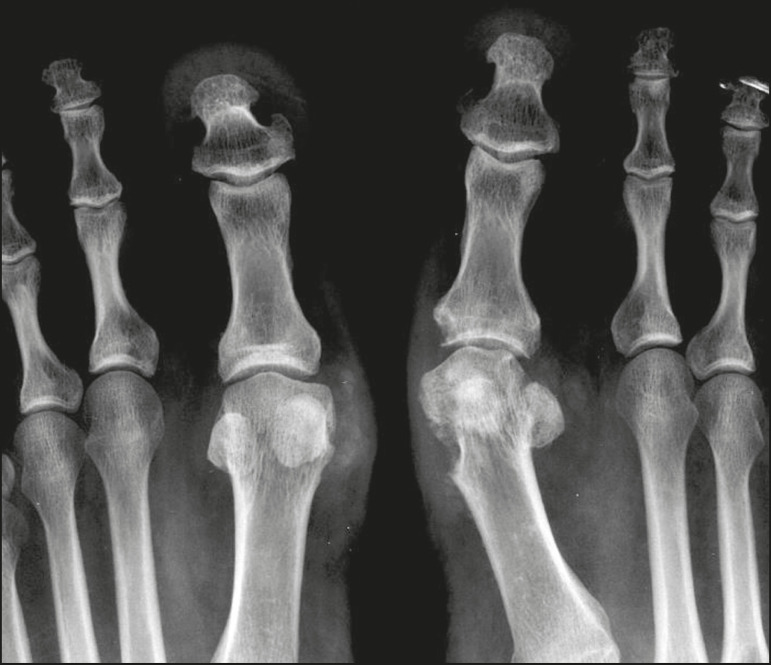
Metastatic calcifications in a gouty tophus. Magnification of an Xray of the feet showing bulging and densification of the subcutaneous tissue in the metacarpophalangeal joint of the hallux, with small foci of calcification, corresponding to a gouty tophus. There are also signs of crystal arthropathy and erosion of cortical bone, especially in the first metacarpophalangeal joint on the left.

Other ionic imbalances, secondary to mechanisms such as skeletal demineralization, massive bone destruction, and increased intestinal absorption, can also cause metastatic calcifications. Gout, which results from hyperuricemia, is one such cause (Figure 14). When calcifications are present in gout, they are usually accompanied by tophi, with or without other bone findings, such as erosion^(2,4,6)^.

## IDIOPATHIC CALCIFICATIONS

**Figure 15:**
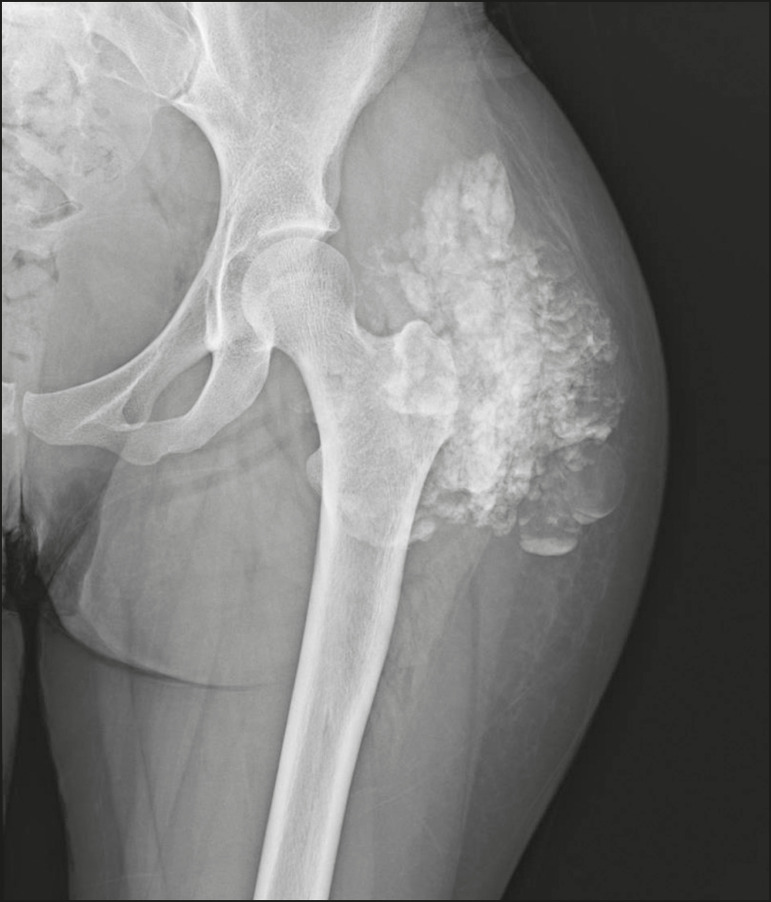
Tumoral calcinosis. Frontal X-ray of the hip showing a calcified peri- calcifications occurring in the thumbs and index fingers. articular mass with lobulated contours and a multicystic appearance in the trochanteric bursa. A fluid-calcium level (the sedimentation sign) can be seen in the cysts.

Idiopathic calcifications occur in tumoral calcinosis, a rare familial disease caused by abnormal regulation of phosphate metabolism. This disease is characterized by the appearance, around the second decade of life, of periarticular calcified masses, which, in imaging tests, are indistinguishable from metastatic calcifications caused by a disorder of calcium and phosphorus metabolism, and are typically distributed on the extensor surfaces (bursal surfaces) of large joints (Figure 15). They are often asymptomatic and can progress slowly. Two forms of idiopathic tumoral calcinosis have been identified^(2,4)^. Although both are caused by specific genetic mutations, phosphate levels are increased in one (the familial form) and normal in the other (the sporadic form). Although metastatic calcifications are often referred to as “secondary tumoral calcinosis”, the term “tumoral calcinosis” should be used strictly in reference to the familial form of the disease^(4)^.

## CALCINOSIS CIRCUMSCRIPTA AND CALCINOSIS UNIVERSALIS

Calcinosis circumscripta and calcinosis universalis are well-established terms, used in order to refer to cutaneous and subcutaneous calcifications that occur in the absence of an underlying metabolic disorder and are typically associated with connective tissue diseases.

**Figure 16:**
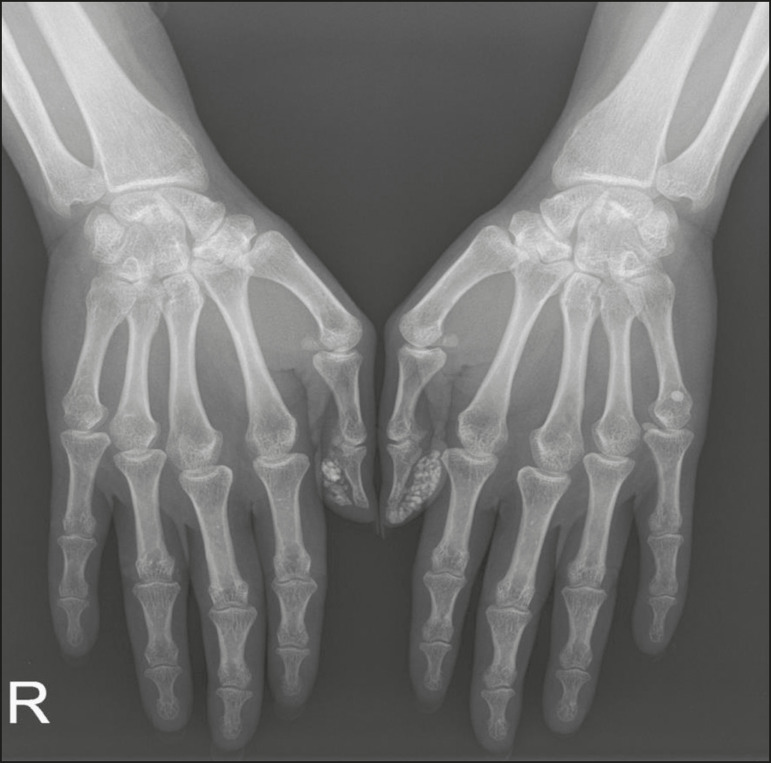
Tumoral calcinosis. Frontal X-ray of the hip showing a calcified peri- calcifications occurring in the thumbs and index fingers. articular mass with lobulated contours and a multicystic appearance in the trochanteric bursa. A fluid-calcium level (the sedimentation sign) can be seen in the cysts.

In calcinosis circumscripta, calcium deposition occurs in a localized way, in the form of densely calcified, homogeneous nodules around the fingertips, especially the thumbs and index fingers^(2)^. Patients develop papules, plaques, and subcutaneous nodules that can ulcerate and discharge a whitish material^(1,4)^. The condition is usually associated with scleroderma^(5)^, as illustrated in Figure 16.

**Figure 17:**
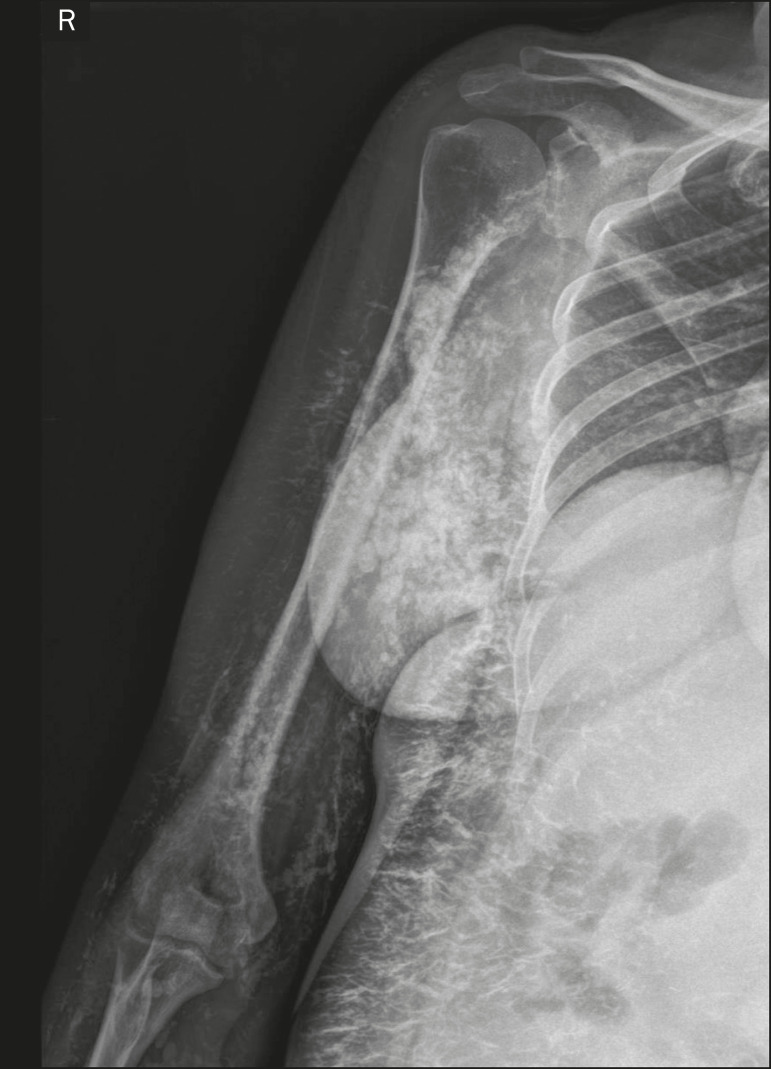
Calcinosis universalis in dermatopolymyositis. Frontal X-ray of the shoulder and arm showing diffuse calcifications in the skin, subcutaneous tissue, fasciae, and muscles. Note the leaf-like distribution of the calcifications.

In calcinosis universalis, there are bands or sheet-like calcifications in subcutaneous, muscle, and fascial tissues with a diffuse, symmetrical distribution. It is usually seen in connective tissue diseases, primarily dermatomyositis and polymyositis^(4)^, as shown in Figure 17.

## CONCLUSION

Soft-tissue calcifications are extremely common findings in imaging tests and are often a source of confusion for radiologists, sometimes prompting unnecessary interventions. A thorough and systematic evaluation of these lesions, in conjunction with the analysis of clinical and biochemical data, can help narrow the differential diagnosis.
